# Training Undergraduates Skills in Breaking Bad News: How Students Value Educators’ Feedback

**DOI:** 10.1007/s13187-018-1415-8

**Published:** 2018-08-21

**Authors:** Marianne Brouwers, Chris van Weel, Roland Laan, Evelyn van Weel-Baumgarten

**Affiliations:** 1 Department of Primary and Community Care, Radboud Institute for Health Sciences, Radboud University Medical Center, Nijmegen, The Netherlands; 2 Department of Health Services Research and Policy, Australian National University, Canberra, Australia; 3 Health Academy, Radboud University Medical Center, Nijmegen, The Netherlands

**Keywords:** Breaking bad news, Communication, Skills training, Feedback, Undergraduate

## Abstract

Feedback is a key factor in acquiring breaking bad news (BBN) communication skills and its’ acceptance depends on the perceived credibility of the provider. Our aim was to investigate students’ opinions on the provided feedback by different educators (surgeons, psychologists, and simulated patient (SP)) during BBN skills training. We developed a questionnaire investigating provided feedback by the surgeon, psychologist, and SP (yes or no statements), regarding (1) perceived safety of the atmosphere, (2) perceived positive feedback, (3) perceived specific feedback, and (4) perceived usefulness for improvement during BBN skills training. Five hundred twenty students returned the questionnaire after BBN skills training. Most students rated the feedback as positive, specific, and useful. Also, the atmosphere was considered safe. Feedback ratings of the SP were the same as for the surgeon and valued higher than for the psychologist. An unsafe atmosphere, or not receiving positive, specific, or useful feedback was mostly related to the psychologist’s feedback. Feedback on BBN skills training by surgeons and SPs is rated equally helpful by students and is regarded specific, useful, and positive. When designing a BBN training, it is worth to consider involving SP’s as well as clinicians.

## Introduction

Feedback is a key factor in acquiring new communication skills [1, 7, 10, 13], thus also for breaking bad news (BBN) skills. It ideally should be provided within a mutual understanding (educational alliance), in which educators and learners both share learning intentions, and should follow the rules of effective feedback to lead to improved performance [4, 12, 14]. However, students often do not remember or recognize when feedback is provided and consistently report it is insufficient [4]. The reception of feedback is influenced by the student’s emotional reaction (e.g., fear) at the time the feedback was provided, the time interval within the feedback is given, or the perceived credibility of the feedback provider [2–4, 6, 8, 15, 16]. Feedback from a high credibility provider (high status, older age, substantial content experience and male gender) improved student performance and satisfaction significantly more than feedback from a low credibility provider [15]. In most medical curricula, teaching communication skills is the domain of the psychologist and includes providing feedback on communication. However, in contrast to doctors, breaking bad news is not their daily professional task, so their content experience regarding BBN is limited and this may filter through when students receive feedback. Also, their perceived status usually is lower [5].

In short, to adequately learn how to break bad news, students need an emotional safe environment, in which highly credible providers can give feedback within a short time frame after practicing it. To our knowledge, no study investigated how students look back on the feedback provided by different educators (surgeons, psychologists, and simulated patients) during communication skills training on how to BBN. Therefore, we compared students’ opinions on the provided feedback by educators in BBN communication skills training. The data had been collected from April 2008 to December 2009, but, due to an unforeseen serious life event of the first author, could only be analyzed after 2016. In order to assess if research since 2009 had made the study less relevant, a Pubmed search (2018) was done of publications on this subject. As no articles could be traced published after 2009, we concluded that our 2009 data would still be relevant for the field and continued with the study regarding this subject.

## Methods

The study took place at the Radboud University Medical Center where students followed a 3-year pre-clinical curriculum of followed by 3 years with clinical clerkships. The curriculum included a longitudinal, helical communication skills program on BBN. Theory and peer-role play in their first and second year preceded the BBN skills training with simulated patients (SP) in a small group sessions (max. 3 students) in their fourth year [17]. All students performed a BBN consultation with an SP (e.g., diagnosis of cancer, and unexpected surgery), while being observed by their peers, psychologist and/or surgeon. Immediately after the performance, students received feedback on their communication skills from the SP, their observing peers, and surgeon or psychologist.

We developed a questionnaire to investigate the feedback. It contained statements regarding the feedback provided by the educators (surgeon, psychologist, and SP). The students were asked to answer the statements by ticking yes or no for each educator separately: whether or not (1) the atmosphere when receiving feedback was safe, (2) positive feedback was provided, (3) the feedback was specific, and (4) the feedback was useful to improve oneself. Students rated the feedback they had received from the SP, surgeon, and/or psychologist when they performed the consultation themselves, as well as the feedback in consultations of their peers. All students participating in the session completed the questionnaire immediately thereafter. Students could, if desired, add additional comments at the end of the questionnaire.

Descriptive statistics (SPSS 22.0) and www.openepi.com were used to calculate percentages and 95% confidence limits for the proportions (Wilson). Questionnaires were analyzed for each statement separately. We excluded responses for a specific statement when (i) a student had not answered that statement for all three educators together and when (ii) a student had rated a statement ambivalent (p.e. circling both yes and no, or added “most of the time”). Additional students’ comments were analyzed with Atlas.ti (version 7.1.5) and quantified to describe frequencies of the categories identified in the analysis.

## Results

Five hundred twenty students (520/591, response rate 88.0%) returned the questionnaire immediately after the BBN skills training. Over 90% of the students rated the provided feedback on their BNN skills from all three educators as positive (96.9%), specific (90.3%), and useful (90.7%). Also, 97.1% of the students reported that the atmosphere when receiving feedback was safe. 0.5% of the students reported that the feedback of either the surgeon or the SP was not positive, while 2.1% of the students reported this for the psychologist’s feedback. Regarding the psychologist’s feedback, students reported an unsafe atmosphere (1.9%) or not receiving specific (5.1%) or useful (3.8%) feedback. Students reported not receiving specific (1.0%) or useful (1.3%) feedback from the surgeon and none of the surgeon’s feedback was reported unsafe. Also, 0.7% of the students reported that the feedback from the SP was unsafe, not specific (2.6%) or not useful (1.5%) (see Table 1).

**Table 1:** Experienced safety and positive feedback, perceived specific feedback and usefulness for improvement by students per educator after BNN skills training

	Surgeon	Psychologist	SP	No of students (%)	95% CI
Safety (*n* = 414)	S	S	S	402 (97.1)	95.0, 98.3
	S	S	US	3 (0.7)	0.2, 2.1
	S	US	S	8 (1.9)	1.0, 3.8
	US	S	S	0	–
	US	US	S	0	–
	US	S	US	1 (0.2)	0.04, 1.4
	S	US	US	0	–
	US	US	US	0	–
Positive feedback (*n* = 419)	P	P	P	406 (96.9)	94.8, 98.2
	P	P	NP	2 (0.5)	0.1, 1.7
	P	NP	P	9 (2.1)	1.1, 4.0
	NP	P	P	2 (0.5)	0.1, 1.7
	NP	NP	P	0	–
	NP	P	NP	0	–
	P	NP	NP	0	–
	NP	NP	NP	0	–
Specific feedback (*n* = 392)	C	C	C	354 (90.3)	87.0, 92.9
	C	C	NC	10 (2.6)	1.4, 4.6
	C	NC	C	20 (5.1)	3.3, 7.7
	NC	C	C	4 (1.0)	0.4, 2.6
	NC	NC	C	1 (0.3)	0.0, 1.4
	NC	C	NC	0	–
	C	NC	NC	3 (0.8)	0.3, 2.2
	NC	NC	NC	0	–
Usefulness for improvement (*n* = 396)	U	U	U	359 (90.7)	87.4, 93.2
	U	U	NU	6 (1.5)	0.7, 3.3
	U	NU	U	15 (3.8)	2.3, 6.2
	NU	U	U	5 (1.3)	0.5, 2.9
	NU	NU	U	1 (0.3)	0.04, 1.4
	NU	U	NU	1 (0.3)	0.04, 1.4
	U	NU	NU	5 (1.3)	0.5, 2.9
	NU	NU	NU	4 (1.0)	0.4, 2.6

*SP*, simulated patient; *S*, safe; *US*, unsafe; *P*, positive; *NP*, not positive; *C*, specific; *NC*, not specific; *U*, useful; *NU*, not useful; *95% CI*, 95% confidence interval score for proportion (Wilson)

Looking at the additional comments, it is important to keep in mind that SPs participated (and provided feedback) in all teaching sessions and the surgeons and psychologists participated and provided feedback in half of the sessions. Of the 520 returned questionnaires, 223 contained additional comments for analysis. In 122 cases, this contained criticism about the experienced feedback. Students reported that the provided feedback was too focused on minor details, difficult or not at all applicable in practice, contradictory, too lengthy, not specific, not safe, too theoretical, too negative, or too much focused on the medical content. Positive comments (69 out of 223) were that the feedback was useful, specific, safe, practical, positive, and careful. Thirty-two comments were neutral (p.e. general comments on breaking bad news education). Further analysis showed that students reported 12 critical comments and 33 positive comments regarding the surgeon’s feedback. They reported 54 critical comments and 7 positive comments regarding the psychologist’s feedback. In addition, students reported 56 critical comments and 29 positive comments regarding the SP’s feedback.

As can be seen in Table 1, although 520 questionnaires were returned less were used for analysis of the statements. Our analysis was intended to show how students rate the educators and their correlations. When students did not rate an educator for a specific statement, that questionnaire was left out for analysis for that specific statement. As some students did not receive or observe feedback from a surgeon or psychologist, those educators are less frequently rated compared to the SP who was always present to give feedback (not in separate table).

## Discussion

This is the first study on how students evaluate feedback by different educators immediately after BBN skills training. The majority of the students was very positive about the provided feedback and valued it as specific and useful for improvement. They also rated the atmosphere as safe.

Acceptance of feedback is subject to the perceived credibility of the feedback provider and this credibility is dependent of professional background and high status, among others [15]. We show that the majority of students rated the feedback provided by the surgeon, psychologist and SP immediately after BNN skills training as positive, specific, and useful for improvement. When looking at the results per educator, the surgeons seem to have received higher ratings for their feedback than the psychologists. This finding is in line with research in the general population in which the status (and thus credibility) of the surgeon is rated higher than status of a psychologist [5]. To our surprise, the feedback ratings for the SP were the same as the ratings for the surgeon (positive feedback and usefulness) and possibly better than the psychologist’s rating. Research tells us that generally, students perceive (simulated) patient feedback as positive [9, 11]. In our opinion, this could be due to a high credibility of the SPs’ feedback on the patient experience for students.

Based on the discipline of the educator, we found that students reported less support and more negative experiences from feedback by the psychologist—in particular, an over-emphasis of negative comments and an unsafe teaching atmosphere. The additional comments mainly reflected and supported the answers of the predefined statements (concerning experienced safety, positive, specific, and useful feedback). This might indicate that students, although they gave their opinion already, find these items of such great importance when receiving feedback during BBN skills training, that they want to reemphasize them. Other comments on the feedback provided included that it was contradictory, too focused on minor details, and too lengthy. Further research is needed to see of these individual comments are shared more in general or only account for a few individuals and to investigate to which educator this can be attributed.

That surgeons (and SPs) seem to carry more convincing authority than psychologists in BBN might also be due to the clinical relevance of the subject and underline that the teaching program managed to simulate a real-life situation. On the other hand, it is possible that personal traits of the educators involved, rather than their discipline, caused this effect.

### Limitations

Our study included all feedback the students had experienced—directed at their performance as well as at the performances they had observed of their peers. In the analysis, it is not possible to distinguish between the two, which could be seen as a limitation. On the other hand, the rationale of the group-based training of BBN is that students will learn from what they observe from their peers, as well as from their own performance.

This study was performed 9 years ago and this raises the question how relevant the data still are 9 years after collection. However, the teaching approach we studied is still used and “state of the art” for BBN teaching. And even more important, we searched the literature and did not find any comparison of feedback between surgeon, psychologist, and simulated patient, not earlier or elsewhere since.

## Conclusion

The majority of the students rate feedback by surgeons, psychologists, and SPs on BBN communication skills as helpful and think the feedback is specific, useful, and positive. However, when looking in detail, the students reported more negative experiences with the psychologist. The feedback of the surgeon and SP were rated equally. Therefore, when designing a BBN-training it is worth to consider involving SPs as well as clinicians in the feedback on BBN communication skills.
